# Supplementary material for: Combining host-derived biomarkers with patient characteristics improves signature performance in predicting tuberculosis treatment outcomes
Source: Commun Biol. 2020 Jul 9;3:359. doi: 10.1038/s42003-020-1087-x (PMC7347567; doi:10.1038/s42003-020-1087-x)
Supplement: Supplementary file 1 — Description of Additional Supplementary Files [file 42003_2020_1087_MOESM1_ESM.pdf]

Description of Additional Supplementary Files

File Name: Supplementary Data 1
Description: Genes, gene names and distributions for the three panels used.

File Name: Supplementary Data 2
Description: Source data used for preparing Figures 2-4.
