# Supplementary material for: Combining host-derived biomarkers with patient characteristics improves signature performance in predicting tuberculosis treatment outcomes
Source: Commun Biol. 2020 Jul 9;3:359. doi: 10.1038/s42003-020-1087-x (PMC7347567; doi:10.1038/s42003-020-1087-x)
Supplement: Supplementary file 3 — Supplementary Data 2 [file 42003_2020_1087_MOESM3_ESM.docx]

Supplementary Data 2: Data used for preparing Figures 2-4

**Figure 2**

Below data for genrating ROC are shown.

Panel A

| ID |  | Basic+Patient | Patient | Basic |
| --- | --- | --- | --- | --- |
|  | Observed | Predicted | Predicted | Predicted |
| 1 | TB Cures | 0.4453 | 0.2147 | 0.4973 |
| 2 | TB Cures | 0.2258 | 0.2167 | 0.3550 |
| 3 | TB Cures | 0.1293 | 0.1932 | 0.2398 |
| 4 | TB Cures | 0.1336 | 0.2072 | 0.3091 |
| 5 | TB Cures | 0.5129 | 0.6520 | 0.3313 |
| 6 | TB Cures | 0.3860 | 0.2240 | 0.4706 |
| 7 | TB Cures | 0.0567 | 0.1010 | 0.2158 |
| 8 | TB Cures | 0.2824 | 0.4353 | 0.3625 |
| 9 | TB Cures | 0.3032 | 0.4066 | 0.3014 |
| 10 | TB Cures | 0.1656 | 0.1345 | 0.2994 |
| 11 | TB Cures | 0.2926 | 0.3093 | 0.3416 |
| 12 | TB Cures | 0.2663 | 0.3842 | 0.3019 |
| 13 | TB Cures | 0.2645 | 0.1673 | 0.3739 |
| 14 | TB Cures | 0.2305 | 0.2152 | 0.3433 |
| 15 | TB Cures | 0.2781 | 0.3920 | 0.3284 |
| 16 | TB Cures | 0.4105 | 0.4383 | 0.4048 |
| 17 | TB Cures | 0.3001 | 0.2265 | 0.4267 |
| 18 | TB Cures | 0.4254 | 0.5180 | 0.3585 |
| 19 | TB Cures | 0.2689 | 0.3290 | 0.3404 |
| 20 | TB Cures | 0.3236 | 0.3808 | 0.3364 |
| 21 | TB Cures | 0.3330 | 0.3290 | 0.3391 |
| 22 | TB Cures | 0.3489 | 0.3135 | 0.4268 |
| 23 | TB Cures | 0.0644 | 0.2138 | 0.1543 |
| 24 | TB Cures | 0.3403 | 0.4080 | 0.3937 |
| 25 | TB Cures | 0.0879 | 0.2130 | 0.1537 |
| 26 | TB Cures | 0.0510 | 0.2273 | 0.1425 |
| 27 | TB Cures | 0.5536 | 0.6257 | 0.4296 |
| 28 | TB Cures | 0.0842 | 0.4882 | 0.1514 |
| 29 | TB Cures | 0.2685 | 0.1055 | 0.4742 |
| 30 | TB Cures | 0.3509 | 0.3449 | 0.3686 |
| 31 | TB Cures | 0.3328 | 0.4376 | 0.3689 |
| 32 | TB Cures | 0.3098 | 0.4355 | 0.3425 |
| 33 | TB Cures | 0.2539 | 0.1494 | 0.3968 |
| 34 | TB Cures | 0.2728 | 0.4313 | 0.2925 |
| 35 | TB Cures | 0.4803 | 0.4369 | 0.4240 |
| 36 | TB Cures | 0.4194 | 0.4034 | 0.4365 |
| 37 | TB Cures | 0.3029 | 0.3372 | 0.3814 |
| 38 | TB Cures | 0.2404 | 0.0985 | 0.4183 |
| 39 | TB Cures | 0.3586 | 0.3014 | 0.4277 |
| 40 | TB Cures | 0.2339 | 0.2929 | 0.3451 |
| 41 | TB Cures | 0.3618 | 0.2275 | 0.4771 |
| 42 | TB Cures | 0.1559 | 0.3261 | 0.2415 |
| 43 | TB Cures | 0.1867 | 0.2191 | 0.2732 |
| 44 | TB Cures | 0.2877 | 0.5452 | 0.2887 |
| 45 | TB Cures | 0.2717 | 0.3939 | 0.3335 |
| 46 | TB Cures | 0.5295 | 0.3943 | 0.4745 |
| 47 | TB Cures | 0.4079 | 0.3290 | 0.4588 |
| 48 | TB Cures | 0.1808 | 0.3109 | 0.2825 |
| 49 | TB Cures | 0.1189 | 0.3488 | 0.1789 |
| 50 | TB Cures | 0.4420 | 0.7180 | 0.2840 |
| 51 | TB Cures | 0.5069 | 0.4257 | 0.4208 |
| 52 | TB Cures | 0.5876 | 0.4355 | 0.5395 |
| 53 | TB Cures | 0.4493 | 0.2937 | 0.4841 |
| 54 | TB Cures | 0.1375 | 0.3228 | 0.2046 |
| 55 | TB Cures | 0.3821 | 0.1657 | 0.5032 |
| 56 | Treatment Failures | 0.3232 | 0.2870 | 0.3819 |
| 57 | Treatment Failures | 0.4436 | 0.3909 | 0.4025 |
| 58 | Treatment Failures | 0.5014 | 0.5346 | 0.3869 |
| 59 | Treatment Failures | 0.3192 | 0.2163 | 0.4078 |
| 60 | Treatment Failures | 0.2960 | 0.2225 | 0.3968 |
| 61 | Treatment Failures | 0.5816 | 0.5251 | 0.3750 |
| 62 | Treatment Failures | 0.4985 | 0.4048 | 0.4224 |
| 63 | Treatment Failures | 0.4317 | 0.5499 | 0.3636 |
| 64 | Treatment Failures | 0.7172 | 0.6481 | 0.5031 |
| 65 | Treatment Failures | 0.4016 | 0.4425 | 0.4036 |
| 66 | Treatment Failures | 0.5545 | 0.7163 | 0.4240 |
| 67 | Treatment Failures | 0.6591 | 0.6335 | 0.4663 |
| 68 | Treatment Failures | 0.7140 | 0.7152 | 0.5096 |
| 69 | Treatment Failures | 0.6688 | 0.6067 | 0.5225 |
| 70 | Treatment Failures | 0.5938 | 0.3255 | 0.5835 |
| 71 | Treatment Failures | 0.5352 | 0.5360 | 0.4551 |
| 72 | Treatment Failures | 0.6261 | 0.5103 | 0.5126 |
| 73 | Treatment Failures | 0.7658 | 0.6455 | 0.6036 |
| 74 | Treatment Failures | 0.4080 | 0.4178 | 0.3314 |
| 75 | Treatment Failures | 0.4616 | 0.6540 | 0.3247 |
| 76 | Treatment Failures | 0.8033 | 0.7322 | 0.5750 |
| 77 | Treatment Failures | 0.6162 | 0.4355 | 0.5171 |
| 78 | Treatment Failures | 0.4923 | 0.3456 | 0.4847 |
| 79 | Treatment Failures | 0.4184 | 0.4482 | 0.3658 |
| 80 | Treatment Failures | 0.3094 | 0.4376 | 0.2918 |
| 81 | Treatment Failures | 0.3550 | 0.2167 | 0.5004 |
| 82 | Treatment Failures | 0.5134 | 0.3857 | 0.4167 |
| 83 | Treatment Failures | 0.6084 | 0.5358 | 0.4792 |
| 84 | Treatment Failures | 0.4924 | 0.4178 | 0.4618 |
| 85 | Treatment Failures | 0.6704 | 0.6291 | 0.4869 |
| 86 | Treatment Failures | 0.5650 | 0.4439 | 0.4829 |
| 87 | Treatment Failures | 0.4892 | 0.3246 | 0.4439 |
| 88 | Treatment Failures | 0.6850 | 0.6317 | 0.5497 |
| 89 | Treatment Failures | 0.6215 | 0.5060 | 0.5014 |
| 90 | Treatment Failures | 0.4638 | 0.3315 | 0.4150 |

Panel B

| ID |  | Basic+Patient | Patient | Basic |
| --- | --- | --- | --- | --- |
|  | **Observed** | **Predicted** | **Predicted** | **Predicted** |
| 1 | TB Cures | 0.1773 | 0.2414 | 0.3835 |
| 2 | TB Cures | 0.0532 | 0.1946 | 0.2560 |
| 3 | TB Cures | 0.1358 | 0.3362 | 0.3361 |
| 4 | TB Cures | 0.0454 | 0.4388 | 0.0572 |
| 5 | TB Cures | 0.0457 | 0.1757 | 0.2117 |
| 6 | TB Cures | 0.0091 | 0.1164 | 0.2957 |
| 7 | TB Cures | 0.0127 | 0.2754 | 0.1112 |
| 8 | TB Cures | 0.1537 | 0.3429 | 0.2332 |
| 9 | TB Cures | 0.1488 | 0.2894 | 0.1732 |
| 10 | TB Cures | 0.1798 | 0.2076 | 0.3427 |
| 11 | TB Cures | 0.3148 | 0.3253 | 0.3580 |
| 12 | TB Cures | 0.1861 | 0.2017 | 0.2761 |
| 13 | TB Cures | 0.3502 | 0.3472 | 0.3536 |
| 14 | TB Cures | 0.0531 | 0.1425 | 0.2739 |
| 15 | TB Cures | 0.0000 | 0.0138 | 0.0543 |
| 16 | TB Cures | 0.2829 | 0.2518 | 0.4001 |
| 17 | TB Cures | 0.0116 | 0.1890 | 0.1250 |
| 18 | TB Cures | 0.0258 | 0.3330 | 0.1475 |
| 19 | TB Cures | 0.0610 | 0.3248 | 0.1528 |
| 20 | TB Cures | 0.0402 | 0.3695 | 0.1398 |
| 21 | TB Cures | 0.1427 | 0.2551 | 0.1664 |
| 22 | TB Cures | 0.1203 | 0.2985 | 0.1928 |
| 23 | TB Cures | 0.0510 | 0.2881 | 0.1333 |
| 24 | TB Cures | 0.2598 | 0.1446 | 0.5815 |
| 25 | TB Cures | 0.1586 | 0.3540 | 0.2063 |
| 26 | TB Cures | 0.2066 | 0.2127 | 0.3732 |
| 27 | TB Cures | 0.2658 | 0.2502 | 0.5262 |
| 28 | TB Cures | 0.2425 | 0.3337 | 0.2942 |
| 29 | TB Cures | 0.0045 | 0.2301 | 0.0622 |
| 30 | TB Cures | 0.0013 | 0.1006 | 0.0789 |
| 31 | TB Cures | 0.0469 | 0.2004 | 0.3274 |
| 32 | TB Cures | 0.1859 | 0.2309 | 0.2301 |
| 33 | TB Cures | 0.0073 | 0.2927 | 0.0407 |
| 34 | TB Cures | 0.0015 | 0.1357 | 0.0541 |
| 35 | TB Cures | 0.4444 | 0.5295 | 0.2916 |
| 36 | TB Cures | 0.1001 | 0.1986 | 0.3921 |
| 37 | TB Cures | 0.2659 | 0.3481 | 0.3801 |
| 38 | TB Cures | 0.2002 | 0.2754 | 0.3280 |
| 39 | TB Cures | 0.0606 | 0.5102 | 0.0369 |
| 40 | TB Cures | 0.4457 | 0.5834 | 0.2661 |
| 41 | TB Cures | 0.3437 | 0.3380 | 0.2948 |
| 42 | TB Cures | 0.1145 | 0.3681 | 0.3019 |
| 43 | TB Cures | 0.3808 | 0.3625 | 0.4140 |
| 44 | TB Cures | 0.1379 | 0.2067 | 0.3629 |
| 45 | TB Cures | 0.2871 | 0.1885 | 0.4302 |
| 46 | Treatment Failures | 0.7158 | 0.3550 | 0.6336 |
| 47 | Treatment Failures | 0.6685 | 0.4820 | 0.5225 |
| 48 | Treatment Failures | 0.7037 | 0.3769 | 0.4842 |
| 49 | Treatment Failures | 0.0635 | 0.1751 | 0.2192 |
| 50 | Treatment Failures | 0.5983 | 0.4682 | 0.3076 |
| 51 | Treatment Failures | 0.7515 | 0.2230 | 0.8038 |
| 52 | Treatment Failures | 0.6603 | 0.4818 | 0.1845 |
| 53 | Treatment Failures | 0.5010 | 0.5768 | 0.1774 |
| 54 | Treatment Failures | 0.4940 | 0.3084 | 0.3703 |
| 55 | Treatment Failures | 0.9794 | 0.7005 | 0.6817 |
| 56 | Treatment Failures | 0.8988 | 0.5158 | 0.5628 |
| 57 | Treatment Failures | 0.8814 | 0.6434 | 0.4306 |
| 58 | Treatment Failures | 0.7393 | 0.4946 | 0.5275 |
| 59 | Treatment Failures | 0.5584 | 0.3815 | 0.2630 |
| 60 | Treatment Failures | 0.9603 | 0.4497 | 0.7782 |
| 61 | Treatment Failures | 0.4175 | 0.3284 | 0.4577 |
| 62 | Treatment Failures | 0.8405 | 0.4529 | 0.5275 |
| 63 | Treatment Failures | 0.3978 | 0.2895 | 0.5197 |
| 64 | Treatment Failures | 0.8156 | 0.4459 | 0.5188 |
| 65 | Treatment Failures | 0.9665 | 0.7488 | 0.5472 |
| 66 | Treatment Failures | 0.7053 | 0.4043 | 0.4170 |
| 67 | Treatment Failures | 0.9200 | 0.3443 | 0.6176 |

Panel C

| ID |  | Basic+Patient | Patient | Basic |
| --- | --- | --- | --- | --- |
|  | **Observed** | **Predicted** | **Predicted** | **Predicted** |
| 1 | TB Cures | 0.2493 | 0.2207 | 0.4029 |
| 2 | TB Cures | 0.1916 | 0.2256 | 0.3476 |
| 3 | TB Cures | 0.4035 | 0.1961 | 0.5052 |
| 4 | TB Cures | 0.1826 | 0.2019 | 0.3280 |
| 5 | TB Cures | 0.5426 | 0.6809 | 0.3750 |
| 6 | TB Cures | 0.2498 | 0.2446 | 0.4381 |
| 7 | TB Cures | 0.2904 | 0.4778 | 0.3078 |
| 8 | TB Cures | 0.4396 | 0.4655 | 0.4671 |
| 9 | TB Cures | 0.1796 | 0.1243 | 0.3430 |
| 10 | TB Cures | 0.2935 | 0.2964 | 0.3605 |
| 11 | TB Cures | 0.1863 | 0.1878 | 0.3589 |
| 12 | TB Cures | 0.2346 | 0.2219 | 0.3954 |
| 13 | TB Cures | 0.3953 | 0.4132 | 0.3172 |
| 14 | TB Cures | 0.4168 | 0.4354 | 0.3876 |
| 15 | TB Cures | 0.2537 | 0.2511 | 0.4304 |
| 16 | TB Cures | 0.4668 | 0.5513 | 0.3824 |
| 17 | TB Cures | 0.4205 | 0.3575 | 0.4695 |
| 18 | TB Cures | 0.3412 | 0.3999 | 0.3518 |
| 19 | TB Cures | 0.3359 | 0.3575 | 0.4103 |
| 20 | TB Cures | 0.2228 | 0.3181 | 0.2836 |
| 21 | TB Cures | 0.2050 | 0.2183 | 0.3570 |
| 22 | TB Cures | 0.3139 | 0.4690 | 0.3337 |
| 23 | TB Cures | 0.2620 | 0.2482 | 0.4085 |
| 24 | TB Cures | 0.1840 | 0.2417 | 0.2850 |
| 25 | TB Cures | 0.6331 | 0.6169 | 0.3832 |
| 26 | TB Cures | 0.2204 | 0.4644 | 0.1990 |
| 27 | TB Cures | 0.1200 | 0.1178 | 0.3651 |
| 28 | TB Cures | 0.3110 | 0.4337 | 0.3045 |
| 29 | TB Cures | 0.5239 | 0.4285 | 0.5202 |
| 30 | TB Cures | 0.1718 | 0.1612 | 0.2979 |
| 31 | TB Cures | 0.3400 | 0.4182 | 0.3188 |
| 32 | TB Cures | 0.3663 | 0.4320 | 0.3363 |
| 33 | TB Cures | 0.3995 | 0.3857 | 0.3650 |
| 34 | TB Cures | 0.3145 | 0.3787 | 0.3807 |
| 35 | TB Cures | 0.1457 | 0.0995 | 0.3837 |
| 36 | TB Cures | 0.3296 | 0.2777 | 0.4211 |
| 37 | TB Cures | 0.2686 | 0.2538 | 0.4250 |
| 38 | TB Cures | 0.2471 | 0.3918 | 0.2895 |
| 39 | TB Cures | 0.2845 | 0.2318 | 0.4353 |
| 40 | TB Cures | 0.5125 | 0.5453 | 0.3859 |
| 41 | TB Cures | 0.3869 | 0.3626 | 0.3541 |
| 42 | TB Cures | 0.5475 | 0.4341 | 0.5697 |
| 43 | TB Cures | 0.4952 | 0.3575 | 0.6100 |
| 44 | TB Cures | 0.3192 | 0.3442 | 0.4095 |
| 45 | TB Cures | 0.3447 | 0.4090 | 0.4488 |
| 46 | TB Cures | 0.6833 | 0.7102 | 0.3806 |
| 47 | TB Cures | 0.4078 | 0.4285 | 0.3730 |
| 48 | TB Cures | 0.2866 | 0.3415 | 0.3617 |
| 49 | TB Cures | 0.1958 | 0.1835 | 0.3651 |
| 50 | Treatment Failures | 0.2914 | 0.2837 | 0.3226 |
| 51 | Treatment Failures | 0.3349 | 0.4255 | 0.3487 |
| 52 | Treatment Failures | 0.5189 | 0.5190 | 0.3905 |
| 53 | Treatment Failures | 0.2272 | 0.2575 | 0.3610 |
| 54 | Treatment Failures | 0.3497 | 0.2407 | 0.5190 |
| 55 | Treatment Failures | 0.6543 | 0.5687 | 0.5531 |
| 56 | Treatment Failures | 0.4177 | 0.3890 | 0.3903 |
| 57 | Treatment Failures | 0.4472 | 0.6278 | 0.3569 |
| 58 | Treatment Failures | 0.6778 | 0.6717 | 0.4688 |
| 59 | Treatment Failures | 0.4178 | 0.4459 | 0.3599 |
| 60 | Treatment Failures | 0.6553 | 0.7059 | 0.3591 |
| 61 | Treatment Failures | 0.7334 | 0.6813 | 0.5425 |
| 62 | Treatment Failures | 0.8188 | 0.7029 | 0.5834 |
| 63 | Treatment Failures | 0.4995 | 0.6048 | 0.2949 |
| 64 | Treatment Failures | 0.5231 | 0.3901 | 0.4770 |
| 65 | Treatment Failures | 0.5288 | 0.5225 | 0.4030 |
| 66 | Treatment Failures | 0.7361 | 0.5189 | 0.6013 |
| 67 | Treatment Failures | 0.7105 | 0.6654 | 0.5295 |
| 68 | Treatment Failures | 0.3648 | 0.4342 | 0.3302 |
| 69 | Treatment Failures | 0.6193 | 0.6855 | 0.4129 |
| 70 | Treatment Failures | 0.7165 | 0.7451 | 0.4581 |
| 71 | Treatment Failures | 0.4806 | 0.4285 | 0.4697 |
| 72 | Treatment Failures | 0.6857 | 0.4005 | 0.5890 |
| 73 | Treatment Failures | 0.6611 | 0.4598 | 0.6881 |
| 74 | Treatment Failures | 0.3567 | 0.4337 | 0.3487 |
| 75 | Treatment Failures | 0.2357 | 0.2256 | 0.3997 |
| 76 | Treatment Failures | 0.7493 | 0.5943 | 0.6476 |
| 77 | Treatment Failures | 0.4475 | 0.4342 | 0.4407 |
| 78 | Treatment Failures | 0.6441 | 0.6252 | 0.4812 |
| 79 | Treatment Failures | 0.3894 | 0.4493 | 0.3867 |
| 80 | Treatment Failures | 0.3356 | 0.3462 | 0.4210 |
| 81 | Treatment Failures | 0.5105 | 0.6318 | 0.3225 |
| 82 | Treatment Failures | 0.5937 | 0.5084 | 0.5059 |
| 83 | Treatment Failures | 0.5503 | 0.3639 | 0.5064 |

Panel D

| ID |  | Basic+Patient | Patient | Basic |
| --- | --- | --- | --- | --- |
|  | **Observed** | **Predicted** | **Predicted** | **Predicted** |
| 1 | TB Cures | 0.2669 | 0.2350 | 0.2495 |
| 2 | TB Cures | 0.2704 | 0.3427 | 0.2634 |
| 3 | TB Cures | 0.2668 | 0.2642 | 0.2549 |
| 4 | TB Cures | 0.2768 | 0.2268 | 0.2886 |
| 5 | TB Cures | 0.2655 | 0.2169 | 0.2412 |
| 6 | TB Cures | 0.2688 | 0.2380 | 0.2549 |
| 7 | TB Cures | 0.2747 | 0.3205 | 0.2863 |
| 8 | TB Cures | 0.2687 | 0.2802 | 0.2670 |
| 9 | TB Cures | 0.2697 | 0.2273 | 0.2577 |
| 10 | TB Cures | 0.2772 | 0.3097 | 0.2923 |
| 11 | TB Cures | 0.2732 | 0.2415 | 0.2742 |
| 12 | TB Cures | 0.2709 | 0.3226 | 0.2699 |
| 13 | TB Cures | 0.2673 | 0.2277 | 0.2495 |
| 14 | TB Cures | 0.2687 | 0.1334 | 0.2543 |
| 15 | TB Cures | 0.2711 | 0.2451 | 0.2640 |
| 16 | TB Cures | 0.2715 | 0.2469 | 0.2680 |
| 17 | TB Cures | 0.2701 | 0.2776 | 0.2606 |
| 18 | TB Cures | 0.2655 | 0.2599 | 0.2406 |
| 19 | TB Cures | 0.2680 | 0.3107 | 0.2510 |
| 20 | TB Cures | 0.2684 | 0.2506 | 0.2564 |
| 21 | TB Cures | 0.2684 | 0.2490 | 0.2551 |
| 22 | TB Cures | 0.2711 | 0.2473 | 0.2720 |
| 23 | TB Cures | 0.2658 | 0.2199 | 0.2432 |
| 24 | TB Cures | 0.2636 | 0.2625 | 0.2329 |
| 25 | TB Cures | 0.2716 | 0.2290 | 0.2701 |
| 26 | TB Cures | 0.2750 | 0.2729 | 0.2834 |
| 27 | TB Cures | 0.2682 | 0.2194 | 0.2513 |
| 28 | TB Cures | 0.2670 | 0.2708 | 0.2478 |
| 29 | TB Cures | 0.2711 | 0.1901 | 0.2826 |
| 30 | TB Cures | 0.2689 | 0.2240 | 0.2562 |
| 31 | TB Cures | 0.2677 | 0.2783 | 0.2493 |
| 32 | TB Cures | 0.2663 | 0.2157 | 0.2472 |
| 33 | TB Cures | 0.2720 | 0.2795 | 0.2755 |
| 34 | TB Cures | 0.2682 | 0.2095 | 0.2518 |
| 35 | TB Cures | 0.2755 | 0.3184 | 0.2838 |
| 36 | TB Cures | 0.2772 | 0.2367 | 0.3047 |
| 37 | TB Cures | 0.2708 | 0.3545 | 0.2791 |
| 38 | TB Cures | 0.2725 | 0.3471 | 0.2916 |
| 39 | TB Cures | 0.2671 | 0.2607 | 0.2515 |
| 40 | TB Cures | 0.2750 | 0.2642 | 0.2799 |
| 41 | TB Cures | 0.2782 | 0.3216 | 0.3172 |
| 42 | TB Cures | 0.2688 | 0.2447 | 0.2538 |
| 43 | TB Cures | 0.2660 | 0.2541 | 0.2517 |
| 44 | Treatment Failures | 0.2643 | 0.2174 | 0.2359 |
| 45 | Treatment Failures | 0.2694 | 0.3211 | 0.2564 |
| 46 | Treatment Failures | 0.2684 | 0.2732 | 0.2533 |
| 47 | Treatment Failures | 0.2790 | 0.3411 | 0.3253 |
| 48 | Treatment Failures | 0.2762 | 0.2272 | 0.3085 |
| 49 | Treatment Failures | 0.2680 | 0.3546 | 0.2506 |
| 50 | Treatment Failures | 0.2764 | 0.3496 | 0.2861 |
| 51 | Treatment Failures | 0.2779 | 0.3553 | 0.3331 |
| 52 | Treatment Failures | 0.2694 | 0.2498 | 0.2616 |
| 53 | Treatment Failures | 0.2932 | 0.3457 | 0.3662 |
| 54 | Treatment Failures | 0.2722 | 0.3625 | 0.2710 |
| 55 | Treatment Failures | 0.2686 | 0.2492 | 0.2539 |
| 56 | Treatment Failures | 0.2736 | 0.2800 | 0.2887 |
| 57 | Treatment Failures | 0.2832 | 0.3841 | 0.3659 |
| 58 | Treatment Failures | 0.2737 | 0.2715 | 0.2986 |
| 59 | Treatment Failures | 0.2703 | 0.2705 | 0.2692 |

**Figure 3**

Below the estimated relative importance, normalized against the variables having the largest importance (this variable has 100%), are shown.

Figure 3, left panel

|  | Variable | Relative importance |
| --- | --- | --- |
| 1 | Gender | 100.0000 |
| 2 | PrevdiagTB | 42.8345 |
| 3 | Tobaccolast6months | 60.3550 |
| 4 | Alcoholpast6months | 42.7499 |
| 5 | Severethinness | 91.2888 |
| 6 | NCAM1 | 1.2645 |
| 7 | CD19 | 25.0010 |
| 8 | AIRE | 52.1427 |
| 9 | NLRP1 | 90.7269 |
| 10 | CXCL13 | 55.9085 |
| 11 | TLR6 | 80.3247 |
| 12 | NLRP2 | 31.1129 |
| 13 | NLRC4 | 21.3562 |
| 14 | TLR8 | 2.5783 |
| 15 | NOD2 | 76.6684 |
| 16 | CXCL10 | 8.8460 |
| 17 | B2M | 2.4986 |

Figure 3, right panel

|  | Variable | Relative importance |
| --- | --- | --- |
| 1 | Age | 1.0952 |
| 2 | Gender | 27.9918 |
| 3 | Cough.2weeks | 19.0200 |
| 4 | Fever | 10.7674 |
| 5 | Tobaccolast6months | 54.1169 |
| 6 | Alcoholpast6months | 5.1392 |
| 7 | BMI | 7.7636 |
| 8 | Severethinness | 26.3359 |
| 9 | BCL2 | 0.2316 |
| 10 | IL13 | 2.9364 |
| 11 | TLR6 | 100.0000 |
| 12 | TLR8 | 0.0017 |
| 13 | CXCL10 | 56.2220 |
| 14 | STAT2 | 11.5400 |

**Figure 4**

Below data for genrating ROC are shown.

Panel A

| ID |  | Basic+Patient | Patient | Basic |
| --- | --- | --- | --- | --- |
|  | **Observed** | **Predicted** | **Predicted** | **Predicted** |
| 1 | Early Responders | 0.7454 | 0.4610 | 0.6464 |
| 2 | Early Responders | 0.4862 | 0.4796 | 0.6445 |
| 3 | Early Responders | 0.2689 | 0.3097 | 0.6441 |
| 4 | Early Responders | 0.0694 | 0.3883 | 0.6417 |
| 5 | Early Responders | 0.6084 | 0.7877 | 0.6446 |
| 6 | Early Responders | 0.4004 | 0.5493 | 0.6461 |
| 7 | Early Responders | 0.0257 | 0.4397 | 0.6369 |
| 8 | Early Responders | 0.6821 | 0.8090 | 0.6449 |
| 9 | Early Responders | 0.4106 | 0.5859 | 0.6459 |
| 10 | Early Responders | 0.3858 | 0.5206 | 0.6442 |
| 11 | Early Responders | 0.5175 | 0.6979 | 0.6425 |
| 12 | Early Responders | 0.2436 | 0.4334 | 0.6452 |
| 13 | Early Responders | 0.4292 | 0.5438 | 0.6470 |
| 14 | Early Responders | 0.2694 | 0.4657 | 0.6448 |
| 15 | Early Responders | 0.1958 | 0.6226 | 0.6421 |
| 16 | Early Responders | 0.7075 | 0.7479 | 0.6434 |
| 17 | Early Responders | 0.4236 | 0.5723 | 0.6457 |
| 18 | Early Responders | 0.4079 | 0.4769 | 0.6450 |
| 19 | Early Responders | 0.5044 | 0.5696 | 0.6443 |
| 20 | Early Responders | 0.0648 | 0.4107 | 0.6443 |
| 21 | Early Responders | 0.2788 | 0.5696 | 0.6440 |
| 22 | Early Responders | 0.1616 | 0.4537 | 0.6436 |
| 23 | Early Responders | 0.1822 | 0.4518 | 0.6427 |
| 24 | Early Responders | 0.1440 | 0.5949 | 0.6441 |
| 25 | Early Responders | 0.0091 | 0.5000 | 0.6416 |
| 26 | Early Responders | 0.2618 | 0.7054 | 0.6427 |
| 27 | Early Responders | 0.5569 | 0.6377 | 0.6431 |
| 28 | Early Responders | 0.1123 | 0.5691 | 0.6431 |
| 29 | Early Responders | 0.3328 | 0.5187 | 0.6444 |
| 30 | Early Responders | 0.3664 | 0.6784 | 0.6450 |
| 31 | Early Responders | 0.3245 | 0.7443 | 0.6430 |
| 32 | Early Responders | 0.4193 | 0.7336 | 0.6425 |
| 33 | Non Responders | 0.8266 | 0.7076 | 0.6456 |
| 34 | Non Responders | 0.6434 | 0.7111 | 0.6431 |
| 35 | Non Responders | 0.8354 | 0.7408 | 0.6450 |
| 36 | Non Responders | 0.9893 | 0.8506 | 0.6453 |
| 37 | Non Responders | 0.7411 | 0.6278 | 0.6458 |
| 38 | Non Responders | 0.7958 | 0.3945 | 0.6448 |
| 39 | Non Responders | 0.8088 | 0.6445 | 0.6451 |
| 40 | Non Responders | 0.8598 | 0.6824 | 0.6444 |
| 41 | Non Responders | 0.7803 | 0.5814 | 0.6432 |
| 42 | Non Responders | 0.7013 | 0.6030 | 0.6445 |
| 43 | Non Responders | 0.7723 | 0.5029 | 0.6453 |
| 44 | Non Responders | 0.8171 | 0.8911 | 0.6434 |
| 45 | Non Responders | 0.9682 | 0.8144 | 0.6453 |
| 46 | Non Responders | 0.8328 | 0.5029 | 0.6408 |
| 47 | Non Responders | 0.6917 | 0.5696 | 0.6457 |
| 48 | Non Responders | 0.8405 | 0.7898 | 0.6445 |
| 49 | Non Responders | 0.5088 | 0.7023 | 0.6446 |
| 50 | Non Responders | 0.8967 | 0.8211 | 0.6437 |
| 51 | Non Responders | 0.6925 | 0.6796 | 0.6446 |
| 52 | Non Responders | 0.8183 | 0.7336 | 0.6461 |
| 53 | Non Responders | 0.9736 | 0.7339 | 0.6470 |
| 54 | Non Responders | 0.4728 | 0.5235 | 0.6432 |
| 55 | Non Responders | 0.7247 | 0.5253 | 0.6451 |
| 56 | Non Responders | 0.5652 | 0.6407 | 0.6435 |
| 57 | Non Responders | 0.5873 | 0.4796 | 0.6441 |
| 58 | Non Responders | 0.8517 | 0.8608 | 0.6439 |
| 59 | Non Responders | 0.8575 | 0.5326 | 0.6460 |
| 60 | Non Responders | 0.4716 | 0.5354 | 0.6451 |
| 61 | Non Responders | 0.9043 | 0.5235 | 0.6448 |
| 62 | Non Responders | 0.9899 | 0.8553 | 0.6430 |
| 63 | Non Responders | 0.7511 | 0.6784 | 0.6450 |
| 64 | Non Responders | 0.9336 | 0.7684 | 0.6460 |
| 65 | Non Responders | 0.6298 | 0.7684 | 0.6444 |
| 66 | Non Responders | 0.8496 | 0.8127 | 0.6446 |
| 67 | Non Responders | 0.9391 | 0.7721 | 0.6455 |
| 68 | Non Responders | 0.9503 | 0.8070 | 0.6443 |
| 69 | Non Responders | 0.8805 | 0.8359 | 0.6439 |
| 70 | Non Responders | 0.9427 | 0.5985 | 0.6454 |
| 71 | Non Responders | 0.9817 | 0.8652 | 0.6455 |
| 72 | Non Responders | 0.8666 | 0.7018 | 0.6444 |
| 73 | Non Responders | 0.9497 | 0.7548 | 0.6457 |
| 74 | Non Responders | 0.8546 | 0.7267 | 0.6449 |
| 75 | Non Responders | 0.9023 | 0.7969 | 0.6435 |
| 76 | Non Responders | 0.9853 | 0.8797 | 0.6458 |
| 77 | Non Responders | 0.9158 | 0.7336 | 0.6470 |
| 78 | Non Responders | 0.8465 | 0.6824 | 0.6454 |
| 79 | Non Responders | 0.8869 | 0.7938 | 0.6435 |
| 80 | Non Responders | 0.5387 | 0.7443 | 0.6446 |
| 81 | Non Responders | 0.7628 | 0.4796 | 0.6447 |
| 82 | Non Responders | 0.6851 | 0.7782 | 0.6436 |
| 83 | Non Responders | 0.6474 | 0.5923 | 0.6456 |
| 84 | Non Responders | 0.9007 | 0.7267 | 0.6438 |
| 85 | Non Responders | 0.9661 | 0.6590 | 0.6449 |
| 86 | Non Responders | 0.8438 | 0.7749 | 0.6470 |
| 87 | Non Responders | 0.8320 | 0.5374 | 0.6452 |
| 88 | Non Responders | 0.9466 | 0.6755 | 0.6448 |
| 89 | Non Responders | 0.7667 | 0.6779 | 0.6443 |
| 90 | Non Responders | 0.8286 | 0.5878 | 0.6470 |

Panel B

| ID |  | Basic+Patient | Patient | Basic |
| --- | --- | --- | --- | --- |
|  | **Observed** | **Predicted** | **Predicted** | **Predicted** |
| 1 | Early Responders | 0.6039 | 0.5284 | 0.7001 |
| 2 | Early Responders | 0.3268 | 0.5412 | 0.5025 |
| 3 | Early Responders | 0.4161 | 0.4448 | 0.6843 |
| 4 | Early Responders | 0.2889 | 0.4769 | 0.5035 |
| 5 | Early Responders | 0.5665 | 0.7572 | 0.4642 |
| 6 | Early Responders | 0.6310 | 0.5887 | 0.6741 |
| 7 | Early Responders | 0.3997 | 0.7327 | 0.2733 |
| 8 | Early Responders | 0.5577 | 0.6134 | 0.6445 |
| 9 | Early Responders | 0.4411 | 0.5337 | 0.5155 |
| 10 | Early Responders | 0.6901 | 0.6828 | 0.6262 |
| 11 | Early Responders | 0.5123 | 0.6218 | 0.7356 |
| 12 | Early Responders | 0.4011 | 0.5316 | 0.5731 |
| 13 | Early Responders | 0.6268 | 0.6147 | 0.6115 |
| 14 | Early Responders | 0.7660 | 0.7277 | 0.7341 |
| 15 | Early Responders | 0.6374 | 0.6042 | 0.6319 |
| 16 | Early Responders | 0.3557 | 0.5501 | 0.5799 |
| 17 | Early Responders | 0.6330 | 0.6127 | 0.7382 |
| 18 | Early Responders | 0.3277 | 0.4930 | 0.5610 |
| 19 | Early Responders | 0.6018 | 0.6127 | 0.6107 |
| 20 | Early Responders | 0.2989 | 0.5341 | 0.4257 |
| 21 | Early Responders | 0.3564 | 0.5220 | 0.5559 |
| 22 | Early Responders | 0.3691 | 0.6195 | 0.4328 |
| 23 | Early Responders | 0.4250 | 0.5824 | 0.5888 |
| 24 | Early Responders | 0.3694 | 0.6737 | 0.2913 |
| 25 | Early Responders | 0.5848 | 0.6507 | 0.5505 |
| 26 | Early Responders | 0.0879 | 0.5939 | 0.1437 |
| 27 | Early Responders | 0.1953 | 0.5949 | 0.3569 |
| 28 | Early Responders | 0.5422 | 0.7251 | 0.4857 |
| 29 | Early Responders | 0.7671 | 0.7174 | 0.7197 |
| 30 | Non Responders | 0.8665 | 0.6658 | 0.7465 |
| 31 | Non Responders | 0.6250 | 0.7014 | 0.5597 |
| 32 | Non Responders | 0.7117 | 0.7226 | 0.7907 |
| 33 | Non Responders | 0.9156 | 0.7570 | 0.9297 |
| 34 | Non Responders | 0.6496 | 0.6516 | 0.5966 |
| 35 | Non Responders | 0.3865 | 0.5090 | 0.5666 |
| 36 | Non Responders | 0.5655 | 0.6455 | 0.5727 |
| 37 | Non Responders | 0.7078 | 0.6103 | 0.6988 |
| 38 | Non Responders | 0.8142 | 0.6603 | 0.8849 |
| 39 | Non Responders | 0.5584 | 0.5572 | 0.6772 |
| 40 | Non Responders | 0.9021 | 0.8068 | 0.6641 |
| 41 | Non Responders | 0.8612 | 0.7223 | 0.5880 |
| 42 | Non Responders | 0.7894 | 0.5572 | 0.8441 |
| 43 | Non Responders | 0.8610 | 0.6127 | 0.8963 |
| 44 | Non Responders | 0.7632 | 0.7074 | 0.5410 |
| 45 | Non Responders | 0.9264 | 0.7023 | 0.9570 |
| 46 | Non Responders | 0.8442 | 0.7369 | 0.6457 |
| 47 | Non Responders | 0.7535 | 0.7174 | 0.6329 |
| 48 | Non Responders | 0.5712 | 0.5817 | 0.7129 |
| 49 | Non Responders | 0.5668 | 0.6096 | 0.6815 |
| 50 | Non Responders | 0.7801 | 0.5908 | 0.7192 |
| 51 | Non Responders | 0.3669 | 0.5412 | 0.5574 |
| 52 | Non Responders | 0.8720 | 0.7749 | 0.6731 |
| 53 | Non Responders | 0.4908 | 0.6042 | 0.5871 |
| 54 | Non Responders | 0.5276 | 0.5793 | 0.6297 |
| 55 | Non Responders | 0.7136 | 0.5817 | 0.8165 |
| 56 | Non Responders | 0.9049 | 0.7617 | 0.6967 |
| 57 | Non Responders | 0.6207 | 0.6859 | 0.6190 |
| 58 | Non Responders | 0.8784 | 0.7427 | 0.7511 |
| 59 | Non Responders | 0.8608 | 0.7427 | 0.7482 |
| 60 | Non Responders | 0.7509 | 0.7294 | 0.5378 |
| 61 | Non Responders | 0.9065 | 0.7014 | 0.8746 |
| 62 | Non Responders | 0.9001 | 0.7243 | 0.8105 |
| 63 | Non Responders | 0.8015 | 0.7425 | 0.5030 |
| 64 | Non Responders | 0.7922 | 0.6574 | 0.7610 |
| 65 | Non Responders | 0.8499 | 0.7794 | 0.6878 |
| 66 | Non Responders | 0.8766 | 0.6856 | 0.8543 |
| 67 | Non Responders | 0.8539 | 0.7328 | 0.7588 |
| 68 | Non Responders | 0.6936 | 0.6652 | 0.5787 |
| 69 | Non Responders | 0.8941 | 0.7643 | 0.8190 |
| 70 | Non Responders | 0.7930 | 0.7945 | 0.5541 |
| 71 | Non Responders | 0.8874 | 0.7174 | 0.8050 |
| 72 | Non Responders | 0.8780 | 0.6886 | 0.7945 |
| 73 | Non Responders | 0.9588 | 0.7619 | 0.9558 |
| 74 | Non Responders | 0.6113 | 0.7251 | 0.5656 |
| 75 | Non Responders | 0.4867 | 0.5412 | 0.6687 |
| 76 | Non Responders | 0.7431 | 0.6279 | 0.7871 |
| 77 | Non Responders | 0.6773 | 0.6652 | 0.5829 |
| 78 | Non Responders | 0.7971 | 0.6652 | 0.7701 |
| 79 | Non Responders | 0.7009 | 0.7476 | 0.6158 |
| 80 | Non Responders | 0.4758 | 0.5911 | 0.6142 |
| 81 | Non Responders | 0.5869 | 0.6766 | 0.5663 |
| 82 | Non Responders | 0.8162 | 0.6687 | 0.7703 |
| 83 | Non Responders | 0.8330 | 0.6249 | 0.8635 |
